# Validation of reference genes for quantitative expression analysis by real-time RT-PCR in *Saccharomyces cerevisiae*

**DOI:** 10.1186/1471-2199-10-99

**Published:** 2009-10-30

**Authors:** Marie-Ange Teste, Manon Duquenne, Jean M François, Jean-Luc Parrou

**Affiliations:** 1 CNRS, UMR5504, F-31400 Toulouse, France; 2 INRA, UMR792 Ingénierie des Systèmes Biologiques et des Procédés, F-31400 Toulouse, France; 3 Université de Toulouse; INSA, UPS, INP; LISBP, 135 Avenue de Rangueil, F-31077 Toulouse, France; 4 Current address: Unité des Bactéries Lactiques et pathogènes Opportunistes, INRA - Centre de Recherche de Jouy-en-Josas, Domaine de Vilvert, 78352 Jouy-en Josas, France

## Abstract

**Background:**

Real-time RT-PCR is the recommended method for quantitative gene expression analysis. A compulsory step is the selection of good reference genes for normalization. A few genes often referred to as HouseKeeping Genes (HSK), such as *ACT1*, *RDN18 *or *PDA1 *are among the most commonly used, as their expression is assumed to remain unchanged over a wide range of conditions. Since this assumption is very unlikely, a geometric averaging of multiple, carefully selected internal control genes is now strongly recommended for normalization to avoid this problem of expression variation of single reference genes. The aim of this work was to search for a set of reference genes for reliable gene expression analysis in *Saccharomyces cerevisiae*.

**Results:**

From public microarray datasets, we selected potential reference genes whose expression remained apparently invariable during long-term growth on glucose. Using the algorithm geNorm, *ALG9*, *TAF10*, *TFC1 *and *UBC6 *turned out to be genes whose expression remained stable, independent of the growth conditions and the strain backgrounds tested in this study. We then showed that the geometric averaging of any subset of three genes among the six most stable genes resulted in very similar normalized data, which contrasted with inconsistent results among various biological samples when the normalization was performed with *ACT1*. Normalization with multiple selected genes was therefore applied to transcriptional analysis of genes involved in glycogen metabolism. We determined an induction ratio of 100-fold for *GPH1 *and 20-fold for *GSY2 *between the exponential phase and the diauxic shift on glucose. There was no induction of these two genes at this transition phase on galactose, although in both cases, the kinetics of glycogen accumulation was similar. In contrast, *SGA1 *expression was independent of the carbon source and increased by 3-fold in stationary phase.

**Conclusion:**

In this work, we provided a set of genes that are suitable reference genes for quantitative gene expression analysis by real-time RT-PCR in yeast biological samples covering a large panel of physiological states. In contrast, we invalidated and discourage the use of *ACT1 *as well as other commonly used reference genes (*PDA1, TDH3, RDN18*, etc) as internal controls for quantitative gene expression analysis in yeast.

## Background

Real-time PCR technology has recently reached a level of sensitivity, accuracy and practical simplicity allowing its use as a routine bioinstrumentation for pathogen detection, single nucleotide polymorphism and gene expression analysis [1-4]. In particular for the latter application, several controls are needed to ensure the integrity of each step along the process [5] and therefore, to obtain reliable and accurate results. This process includes RNA extraction (yield, integrity, DNA contamination), efficiency of the reverse transcription and PCR steps, amount of RNA added into the reaction, etc. While the quantitative RT-PCR is technically robust, the normalization procedure to correct sample-to-sample variation remains a critical and challenging problem of this method [1,4,6,7]. Several procedures have been suggested based on physical parameters, such as volume or cell number, but these methods are either impractical or unreliable due to the heterogeneity of biological samples. Some authors favour an internal control strategy, which uses an alien RNA molecule that is artificially incorporated into the biological sample [7]. As an example, Liu & Slininger proposed a set of universal external RNA calibrators for microbial mRNA expression analysis [8]. In spite of these initiatives, the most common practice is to normalize to either total RNA amount, ribosomal RNA or to a single internal reference gene termed HouseKeeping gene (HSK). Several mathematical models have been developed that calculate the "relative" mRNA expression changes of a target gene with respect to an HSK. The "2ΔΔCt" approach [9] is the most popular application in quantitative RT-PCR, but it assumes optimal and identical PCR efficiencies of target and reference genes. Violation of this rule results in a systematic bias that either underestimates or overestimates the initial copy numbers. This problem can be bypassed by adjusting for PCR efficiency, which can be estimated using many approaches [10-12] that can be separated into three groups [12]: serial dilutions, individual graph analysis based on the rate of fluorescence accumulation within the exponential region, or mathematical model fitting. Whatever the method employed for determining the PCR efficiency, accurate relative quantification implies that the expression of the reference gene is perfectly stable in the sample set.

It is empirically assumed that housekeeping genes fulfil the criterion of unregulated expression independent of the experimental condition. However, some evidence shows that these genes are regulated to some extent, reinforcing the idea that there is no universal reference gene whose expression level remains constant whatever the conditions [2]. Since even small variations of an internal control could lead to non-reliable expression data, it is critical to validate that the expression of reference genes is stable prior to their use for normalization in real time RT-PCR analysis. To overcome the "circular problem" of evaluating the expression stability of a candidate gene if no reliable measure is available to normalize the candidate [13], Vandesompele and colleagues [14] developed a statistical algorithm termed geNorm. Their strategy relied on (i) a careful selection of a set of genes that display minimal variation across different biological conditions, and (ii) normalization of the genes of interest to the geometric mean of a minimal, albeit optimal number of the selected genes. The strength of using geometric averaging is in smoothing the individual variation of the expression value of a single reference gene, which can lead to large errors of normalized data in samples of interest [14]. Other statistical algorithms were also proposed, *e.g*. Bestkeeper [15], which allows including up to ten genes of interest in the analysis, or Normfinder [13] that is apparently less sensitive toward coregulation of the candidate reference genes.

Real-time RT-PCR is commonly used to validate microarray-generated data [16,17]. *ACT1 *and *RDN18 *are among the most frequently used reference genes in *S. cerevisiae *studies, because the expression of these genes has been considered relatively stable under the conditions investigated. However, only two recent papers showed the stability of *ACT1 *expression and some other standard reference genes to normalize the expression of genes involved in central carbon metabolism during short-term glucose pulse [18], or during the rehydration process in active dry yeast [19]. With the notable exception of these works, we could not find any study dedicated to the selection and validation of suitable reference genes in *S. cerevisaie*, contrary to other fungal models such as the pathogenic yeast *Candida albicans *[20], and the fungi *Metarhizium anisopliae *[21] and *Aspergillus niger *[22]. Therefore, the purpose of the present work was to identify a robust set of reference genes for growth phase-related mRNA profiling in the yeast *Saccharomyces cerevisiae*. From public microarray datasets, we selected a set of potential reference genes that exhibited minimal variation among various conditions. The most stable subset of internal controls, which gave rise to a robust normalization factor, was then applied to quantify expression of genes involved in glycogen metabolism in response to changing growth conditions, and in a mutant defective in *TPS1 *which encodes the trehalose-6P synthase subunit [23].

## Results

### Sampling

**Figure 1:**
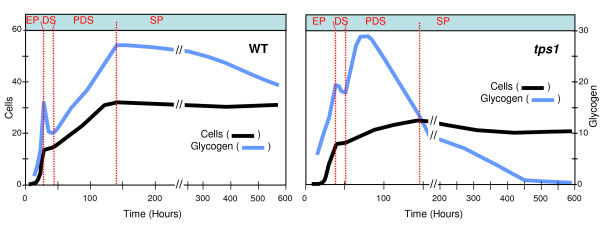
**Schematic view of growth characteristics of a WT strain and its *tps1 *derivative**. Growth (cells) and glycogen profiles during cultures of CEN.PK strains on galactose. WT (left, set D from Figure 2) and *tps1 *(right, set H from Figure 2). EP, Exponential Phase; DS, Diauxic Shift; PDS, Post-Diauxic Shift; SP, Stationary Phase. Original data and sampling numbering can be found in the Additional files. Cells (OD_600_), Glycogen (μg eq.glucose/OD unit).

**Figure 2:**
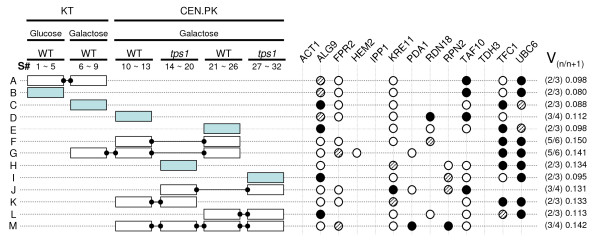
**Sample sets and Ranking of candidate reference genes as calculated by geNorm**. Left panel: Independent cultures (illustrated by the boxes) were carried out: Wild type KT strain on glucose (sample set B) and galactose (set C); Wild type CEN.PK (set D & set E as independent cultures) and its *tps1 *derivative strain (set H & set I as independent cultures), on galactose. Sampling [S#] was performed all along the cultures with a posteriori selection and analysis of 4 to 7 RNA samples representative of different physiological states (e.g. samples 1 to 5 for growth of the KT strain on glucose; see Additional file 1). Expression data from one culture (e.g. set B/) or from several cultures (connector between boxes, e.g. set A/that includes samples from sets B & C together) were then analyzed with geNorm (A ~M sample sets). Right panel: Synthetic overview of ranking of the candidate reference genes according to their expression stability, and determination of the optimal number of genes used for normalization. The 2 most stable genes (black circle), the third (dashed circle) and the following 3 best reference genes (empty circle). Pair-wise variation (V_n/n+1_) between NF_n _and NF_n+1 _(NF: normalization factor; n: number of genes used for NF calculation). Right Column: pair-wise variation value below the threshold 0.15, which means that n genes might be sufficient for NF calculation (i.e. 2 genes for set "A"). See additional file 3 for overall stability under the standard geNorm output format.

Cell samples were regularly harvested from the yeast cultures, and only samples from key physiological states were selected and used for mRNA quantification by real time RT-PCR assays (Figure 1 and Additional files 1 and 2). These physiological states were defined from the macrokinetic growth parameters and reserve carbohydrates profiles as follows: the exponential - respiro fermentative- phase (EP); the diauxic shift (DS), which corresponds to the time when the sugar has just been exhausted from the medium while glycogen shows a transient peak of accumulation; the post-diauxic or purely respiratory phase (PDS), which corresponds to the re-assimilation of fermentation products and to a second phase of glycogen accumulation; and finally, the stationary phase (SP) when cells are starved for carbon nutrient. Contrary to the wild type strain behaviour, the *tps1 *mutant significantly mobilized glycogen during the PDS phase, and consequently more samples were taken up during this period to better characterize the physiological state of this mutant in this growth phase (see also additional file 2 for details of the sampling). In total, we carried out six independent yeast cultures (Figure 2). The wild type KT strain was grown on glucose (sample set B) as the basic and reference growth condition [24]. It was also cultivated on galactose (set C), which was used as the growth control condition for *tps1 *mutant since this mutant strain cannot grow on glucose [25]. Finally, cultures on galactose of the CEN.PK strain and the corresponding *tps1 *mutant were made in duplicates (sets D & E for the wild type CEN.PK and sets H & I for the *tps1 *mutant).

### Expression level and stability of candidate reference genes

As stated in the introduction, accurate normalization requires reference genes whose expression changes are negligible under the investigated conditions. Candidate genes were therefore identified using public microarray datasets from De Risi *et al *[26] and Gasch *et al *[27], because the culture conditions reported in these studies were the closest to our experimental setup. We selected eight potential reference genes based on the stability of their expression during growth on glucose (genes highlighted in bold in Table 1), taking care that these genes belong to different functional categories to minimize the risk of coregulation. The remaining genes listed in Table 1, *i.e. ACT1*, *PDA1*, *RDN18*, *IPP1 *and *TDH3*, were also included in the list since they are traditionally used as single reference genes in expression studies by Northern blots or real time RT-PCR.

**Table 1:** List of candidate reference genes and genes of interest

**Name**	**Molecular Function (SGD curated)/Biological process**	**Primer sequence**	**Eff**
**Candidate reference genes**			

*ACT1*	Structural constituent of cytoskeleton/Cell polarization, endocytosis, and other cytoskeletal functions	F:ATTATATGTTTAGAGGTTGCTGCTTTGG R:CAATTCGTTGTAGAAGGTATGATGCC	94

***ALG9***	Mannosyltransferase activity/Protein amino acid glycosylation	F:CACGGATAGTGGCTTTGGTGAACAATTAC R:TATGATTATCTGGCAGCAGGAAAGAACTTGGG	93

***FPR2***	Membrane-bound peptidyl-prolyl cis-trans isomerase activity/Unknown	F:TCTTTATTAGAATCGGGAACTGTATTTGACTC R:AATGACGCCTGGGACACCTCTTTC	89

***HEM2***	Porphobilinogen synthase activity/Heme biosynthesis	F:TTCCGCTATTCATCTCCGATAATCCAG R:ACAGACATCGCAAATAATATACAGTTCAGG	95

*IPP1*	Inorganic diphosphatase activity/Phosphate metabolic process	F:CCCAATCATCCAAGACACCAAGAAGG R:AGCAATAGTTTCACCAATTTCCAACACATC	90

***KRE11***	Unknown/ER to Golgi vesicle-mediated transport	F:AACTGGTTCTGTTACCCAAATCAACTCAAC R:AACGCTTCAATGTGACTTCTGTTTCCC	86

*PDA1*	Pyruvate dehydrogenase activity/Pyruvate metabolism	F:ATTTGCCCGTCGTGTTTTGCTGTG R:TATGCTGAATCTCGTCTCTAGTTCTGTAGG	93

*RDN18*	Sstructural constituent of ribosome/Translation	F:AACTCACCAGGTCCAGACACAATAAGG R:AAGGTCTCGTTCGTTATCGCAATTAAGC	93

***RPN2***	Protein binding, bridging/Ubiquitin-dependent protein catabolic process	F:GCGGATACAGGCACATTGGATACC R:TGTTGCTACCTTCTCTACCTCCTTACC	101

***TAF10***	RNA Pol II transcription factor activity/Transcription initiation and chromatin modification	F:ATATTCCAGGATCAGGTCTTCCGTAGC R:GTAGTCTTCTCATTCTGTTGATGTTGTTGTTG	96

*TDH3*	Glyceraldehyde-3P dehydrogenase (phosphorylating) activity/Glycolysis & Gluconeogenesis	F:CGGTAGATACGCTGGTGAAGTTTC R:TGGAAGATGGAGCAGTGATAACAAC	91

***TFC1***	RNA Pol III transcription factor activity/Transcription initiation on Pol III promoter	F:GCTGGCACTCATATCTTATCGTTTCACAATGG R:GAACCTGCTGTCAATACCGCCTGGAG	91

***UBC6***	Ubiquitin-protein ligase activity/ER-associated protein catabolic process	F:GATACTTGGAATCCTGGCTGGTCTGTCTC R:AAAGGGTCTTCTGTTTCATCACCTGTATTTGC	84

**Genes of interest (GOIs) in glycogen metabolism**			

*GPH1*	Glycogen phosphorylase activity/Glycogen catabolic process	F:ACAAAACTCAGCAGAAATTCACCACAAG R:CAAGACGACCTAGACCACCATTACC	90

*GSY2*	Glycogen synthase activity/Glycogen biosynthetic process	F:TGCCCAGTATAAAGACCATTACCACTTGATAGG R:GCACCTTCAATCAGCCACCTCCCATAAAC	86

*SGA1*	glucan 1,4-alpha-glucosidase activity/Glycogen catabolic process	F:TCCAAACGGATATTTCCTGGGTGGTACTGAG R:GCATGATCTATTGTGTTTACATTAGCGGGTAG	89

Function of candidate reference genes and GOIs as annotated in the SGD database . All ORF sequences were recovered from the SGD database. Forward (F) and reverse (R) primer sequences; PCR amplification efficiency (Eff). Genes highlighted in bold were selected from their apparent stability during growth on glucose (public microarray datasets from De Risi *et al *[26] and Gasch *et al *[27]). The remaining reference genes are commonly used internal controls in yeast studies.

**Figure 3:**
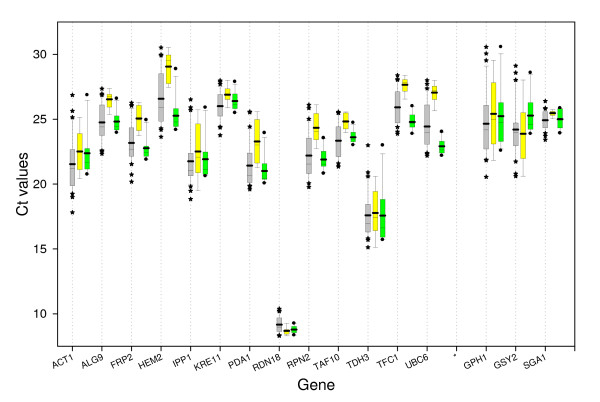
**Distribution overview of expression levels (Ct) of the different genes**. Boxplot representation of raw Ct values obtained from amplification curves. Lower and upper boundaries of the box indicate the 25th and the 75th percentile, respectively, the thin line within the box marks the median, and the whiskers (error bars) below and above the box indicate the 10th and 90th percentiles. Mean (thick line) and outliers (*). Complete RNA sample set from the study (n = 32, grey), sample set "A" (n = 9, yellow; see Figure 2, Glucose + Galactose) and sample set "K" (n = 11, green; see Figure 2, WT + *tps1Δ*). As stated in methods, the 25 μL reaction mixes contained 5 μL of cDNA preparation diluted 10 times, except for *RDN18 *where cDNA was diluted 50 times. For a easy and preliminary estimation of the relative expression of a gene between two samples, a difference of 3.33 Ct with 100% PCR efficiency represents 10-fold over-expression or repression between two conditions (N_2_/N_1 _= (1+Eff)^(Ct_1_-Ct_2_)); With PCR efficiency correction, the same Ct difference with only 90% efficiency signifies a 8.5-fold variation of transcripts between the two samples.

Transcription profiling using real-time RT-PCR assays was then performed with these 13 candidate genes, in samples from the 6 cultures. We first analyzed transcript abundance of these genes in the different samples by direct comparison of their cycle threshold (Ct), assuming equal Ct for equal transcript number since all RT-PCR reactions were performed with equal quantity of total RNA. As can be seen in Figure 3, most of the selected genes presented Ct values that spanned from 20 to 30 cycles, while Ct values from *RDN18 *and *TDH3 *were clearly lower. For *RDN18*, these values centered around 8 cycles with a very low dispersion. The glycolytic gene *TDH3 *was also highly expressed as indicated by Ct values around 17 cycles, but it exhibited rather high dispersion over the growth phases and culture conditions as indicated by large whiskers of the box and many outliers. The Ct of the remaining selected genes showed a reasonable dispersion, with expression levels of *ALG9*, *KRE11*, *TAF10*, *TFC1 *and *UBC6 *exhibiting smaller variation than that of *ACT1*, *HEM2*, *IPP1 *or *PDA1*.

These raw Ct data were then analyzed using geNorm to identify the most suitable candidate genes. For each independent culture (*e.g*. sample set B, Figure 2 left panel), or pool of several cultures (*e.g*. set A that combined samples from cultures B & C), the 13 genes were ranked according to their gene expression stability measure "M" (Figure 2, right panel, and additional file 3). All genes presented an M value below 1.5, which is the default limit for acceptable expression stability as defined by Vandesompele *et al *[14]. Another advantage of geNorm is to provide the optimal number of reference genes required for accurate normalization. This number is obtained by calculating the Pairwise variation values (V_(n/n+1)_) between each combination of sequential normalization factors (NF) (Figure 2, right column). Vandesompele and coworkers [14] recommended a cut-off value at 0.15, below which the inclusion of an additional gene does not result in a significant improvement of the normalization. According to this criterion, *TAF10 *and *UBC6 *turned out to be sufficient as internal controls to normalize expression levels from samples taken from growth on glucose (set A, V_2/3 _= 0.098), whereas 5 genes were required for normalizing gene expression from the data sets F and G (V_5/6 _= 0.150 and 0.141, respectively). According to the recommendation of Vandesompele *et al *[14], we always used a minimum of three of the most stably expressed genes to calculate the normalization factor. From this analysis, *ACT1*, *IPP1 *and *TDH3 *were excluded from the set of selected genes for normalization as they always ranked among the worst candidates. In contrast, *ALG9*, *TAF10*, *TFC1*, *UBC6 *and to a lesser extent *KRE11 *turned out to be the most stable genes in the culture conditions tested in this study (Figure 2 right panel, additional file 3).

**Figure 4:**
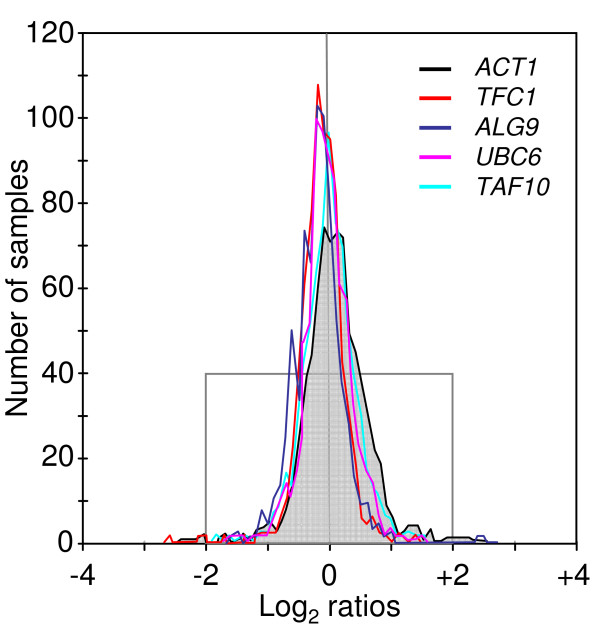
**Expression summary as reported in the SGD Expression Connection tool**. For each gene (depicted by different color lines), the pattern that is reported in this figure is a copy of the "expression summary" histogram that was obtained using the Expression Connection tool from the SGD server . This graph indicates the number of samples (also called 'experiments' on the SGD server), at a given expression ratio value, that could be found in all the microarray datasets stored on the SGD server (*i.e. approx*. 30 studies). The expression data reported on the axis are in log2 scale.

To further support this conclusion and the suitability of this set of genes to serve as a reference in a broader panel of experimental conditions, we examined gene stability of these candidate reference genes by using data from the entire microarray datasets from the SGD server (*approx*. 30 experiments), which altogether correspond to several hundred different experimental conditions. As can be seen in Figure 4, genes like *ALG9*, *TAF10*, *TFC1*, *UBC6 *presented a significantly higher number of experiments with a log2 ratio close to zero as compared to *ACT1*. This microarray survey analysis indicated that our initially selected genes exhibited very little expression change over a wide range of experimental conditions. Therefore, this set of genes, *ALG9*, *TAF10*, *TFC1 *and *UBC6 *should be preferentially used to calculate normalization factors in quantitative RT-PCR expression analysis in the yeast *S. cerevisiae*.

### Impact of reference gene selection on expression ratio values

**Figure 5:**
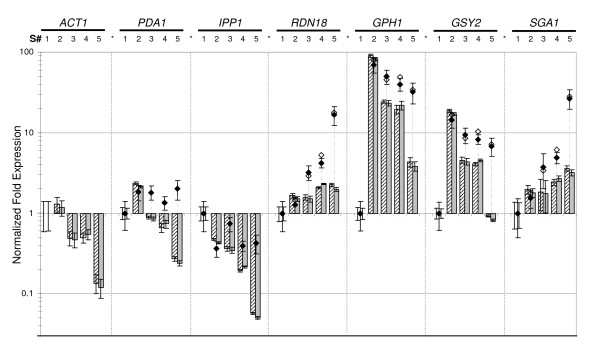
**Effect of normalization strategies on expression ratios**. Normalized expression of *ACT1, PDA1, IPP1, RDN18, GPH1*, *GSY2 *and *SGA1*, in 5 characteristic samples during growth on glucose (*i.e*. set "B" in Figure 2): early exponential phase (respiro-fermentative), entry in (disappearance of glucose) and exit from the diauxic shift, mid of post-diauxic (respiratory) growth, and 3 days stationary phase. The exponential phase sample (S#1) was used as calibrator. Normalization was performed using the three most stable genes (NF_(*UBC*6, *TAF*10, *ALG*9)_, dashed bar), the following 3 best (NF_(*TFC*1, *KRE*11, *FRP*2)_, grey bar), *ACT1 *alone (NF_(*ACT*1)_, black diamond) or using *ACT1, PDA1 *and *IPP1 *(NF_(*ACT*1, *PDA*1, *IPP*1)_, empty diamond). Normalized expression data and error bars were calculated using the gene expression module of the BIORAD iQ5 software, which follows models and error propagation rules outlined in the geNorm manual. For the sake of clarity, we did not plot standard deviation of ratios obtained from NF_(*ACT*1, *PDA*1, *IPP*1)_.

The strength of GeNorm to select the most suitable reference genes was demonstrated by comparing normalized data calculated from different subsets of potential HSKs. As is shown in Figure 5, the use of a normalization factor based on the geometric mean of expression levels of *UBC6*, *TAF10 *and *ALG9 *(NF_(*UBC*6, *TAF*10, *ALG*9)_) yielded expected expression patterns of the glycogen metabolic genes *GSY2 *and *GPH1 *during growth of the KT strain on glucose (see last results section for more details). Ratios of expression values were almost identical using the following 3 best genes based on geNorm classification (NF_(*TFC*1, *KRE*11, *FRP*2)_, compare grey and hatched bars) or applying the normalization factor calculated from the six best genes together (NF_(6 best)_, not plotted). These results showed that, at least in this condition, any subset of three genes among the most stably expressed candidates was sufficient to calculate robust NF and to normalize expression of Genes of Interest (GOIs).

**Figure 6:**
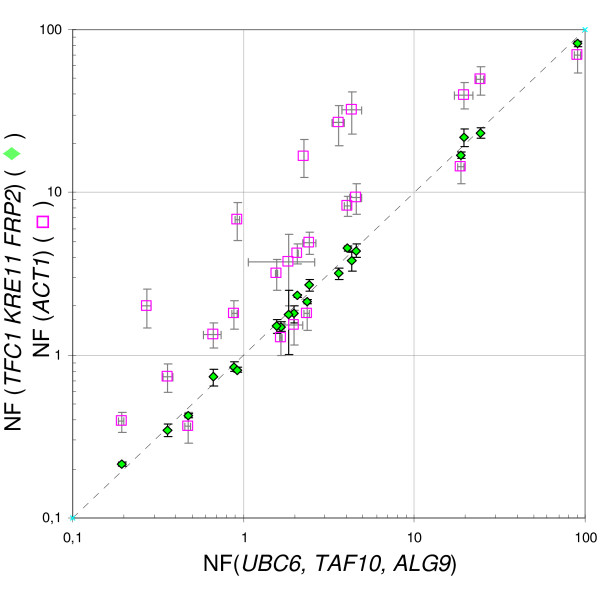
**Degree of correlation between normalization strategies in simple datasets**. Scatter plot of the data illustrated in Figure 5. X axis: ratios calculated using the three most stable genes (NF_(*UBC*6, *TAF*10, *ALG*9)_); Y axis: ratios calculated using NF_(*TFC*1, *KRE*11,* FRP*2) _(green diamond) or NF_(*ACT*1) _(purple square). Horizontal and vertical error bars: Standard deviation on X and Y ratio, respectively. Grey Dotted line: y = x. The equation and correlation coefficient of the linear regression fit (not reported) are y = 0,9178x, R^2 ^= 0,997 (green diamonds), and y = 0,9327x, R^2 ^= 0,598 (purple squares).

The advantage of using validated genes for normalization was further analyzed comparing expression results after normalization (NF_(*UBC*6, *TAF*10, *ALG*9)_, etc) to those obtained by using non-validated reference genes, *e.g. ACT1 *(NF_(*ACT*1)_) or a combination of *ACT1*, *PDA1 *and *IPP1 *(NF_(*ACT*1, *PDA*1, *IPP*1)_). As it could be expected from the coregulation of these three genes during growth on glucose (Figure 5), identical expression data were obtained with NF_(*ACT*1) _and NF_(*ACT*1, *PDA*1, *IPP*1) _as normalization factors (compare empty and black dots for *RDN18 *and GOIs normalized expression data). In contrast, a strong discrepancy between normalization to *ACT1 *and normalization to validated genes was observed in biological samples collected in the post-diauxic phase (#3 and #4) and in the stationary phase (#5). This strong deviation could be explained by the drop of *ACT1 *mRNA as well as that of transcripts of other HSK genes (*IPP1 *and *PDA1*) during these growth phases (Figure 5). To better visualize the advantage of using normalization to validated genes, data from sample set B were reported on a scatter plot (Figure 6), comparing data normalized to NF_(*TFC*1, *KRE*11, *FRP*2) _and NF_(*ACT*1)_, respectively, to those normalized to NF_(*UBC*6, *TAF*10, *ALG*9) _(Figure 6). A regression coefficient close to one (R^2 ^= 0.997) was calculated for NF_(*TFC*1, *KRE*11, *FRP*2) _*versus *NF_(*UBC*6, *TAF*10, *ALG*9)_. In contrast, the coefficient was extremely low for NF_(*ACT*1) _*versus *NF_(*UBC*6, *TAF*10, *ALG*9) _(R^2 ^= 0.598), mainly due to expression data from PDS and SP samples.

**Figure 7:**
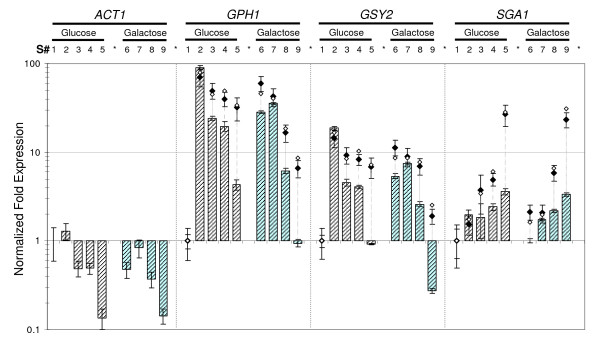
**Effect of carbon source on expression profiles of the commonly-used *ACT1 *and genes of interest**. Normalized expression of *ACT1 *and *GPH1, GSY2, SGA1 *in sample set "A" (n = 9). This set includes 5 samples selected during growth on glucose (grey, see legend from Figure 5) and 4 characteristic samples from growth on galactose (blue): early exponential phase (respiro-fermentative), entry in the diauxic shift (disappearance of galactose), mid of post-diauxic (respiratory) growth, and 3 days stationary phase. The exponential phase sample on glucose was used as calibrator for this sample set. Normalization was performed using the three most stable genes (NF_(*UBC*6, *TAF*10, *ALG*9)_, dashed bar), the geometric mean of *ACT1, PDA1 *and *IPP1 *(NF_(*ACT*1, *PDA*1, *IPP*1)_, empty diamond) or *ACT1 *alone (NF_(*ACT*1)_, black diamond). Normalized expression data and error bars calculated as described in Figure 5. For the sake of clarity, we did not plot standard deviation of ratios obtained from NF_(*ACT*1, *PDA*1, *IPP*1)_.

**Figure 8:**
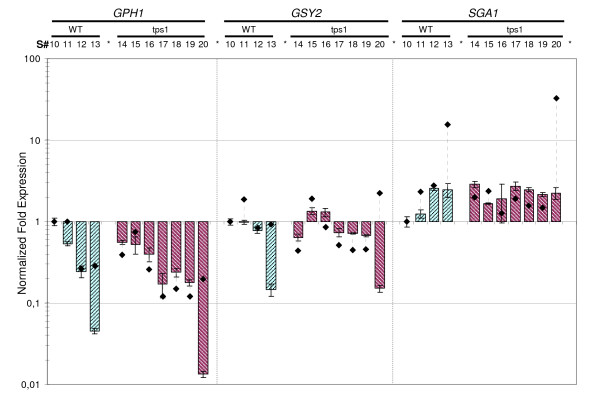
**Effect of *tps1 *mutation on expression profiles of genes of interest**. Normalized expression of *GPH1, GSY2 *and *SGA1 *in WT and *tps1Δ *strains grown on galactose (set "K"). This set includes i/4 samples selected during growth of the WT strain (blue): early exponential phase [respiro-fermentative], exit from the diauxic shift, mid of post-diauxic [respiratory] growth, and after 3 days in stationary phase; and ii/7 samples from growth of the *tps1Δ *strain (red): early exponential phase [respiro-fermentative], entry in and exit from the diauxic shift, early and mid respiratory growth (i.e. just before and just after glycogen peak), entry in and after 3 days in stationary phase. The exponential phase sample of the WT strain was used as calibrator. Normalization was performed using the three most stable genes in this sample set (NF_(*UBC*6, *TFC*1, *KRE*11)_, dashed bar) or *ACT1 *alone (NF_(*ACT*1)_, black diamond). Normalized expression data and error bars calculated as described in Figure 5. For the sake of clarity, we did not plot standard deviation of ratios obtained using *ACT1 *as reference.

**Figure 9:**
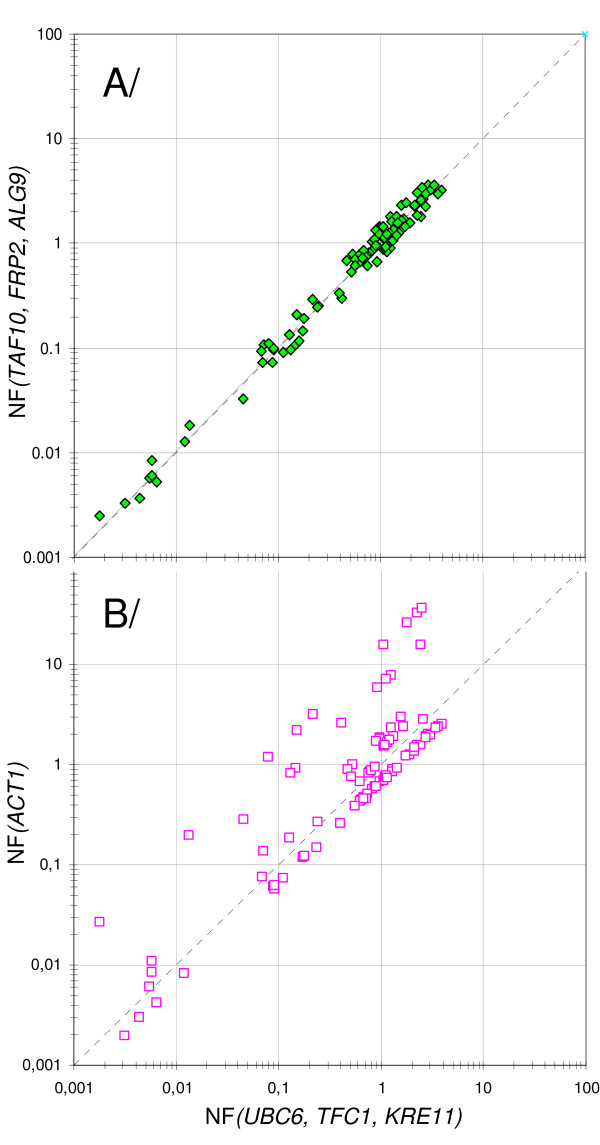
**Degree of correlation between normalization strategies in more heterogeneous datasets**. Scatter plot of ratio values obtained from sample set "K" for genes which together illustrated a wide range of responses (from strong over-expression to repression, see Figure 8 for some of them). X axis: ratios calculated using the three most stable genes (NF_(*UBC*6, *TFC*1, *KRE*11)_); Y axis: ratios calculated using NF_(*TAF*10, *FRP*2, *ALG*9) _(A) and NF_(*ACT*1) _(B). Dotted line: y = x. The equation and correlation coefficient of the linear regression fit (not reported) were y = 1.030x, R^2 ^= 0.916 (A) and y = 2.405x, R^2 ^= 0.124 (B).

Similar analyses were carried out by using different biological situations, as for instance, in a biological set combining samples from cultures on glucose and galactose to analyze the influence of the carbon source (Figure 7, set A), or from wild type and *tps1 *mutants to analyze the impact of the mutation on the expression data (Figure 8, set K). Again, discrepancies in ratios of expression values calculated by normalization to the "best reference genes" and to *ACT1*, respectively, were evident in samples collected from yeast cultures entering the diauxic shift. The difference was even more pronounced with late stationary phase samples, as the difference could reach almost 10-fold between the two procedures (see Figure 7 &8). This discrepancy was visualized in the scatter plots presented in Figure 9, which report a larger range of ratio values than those in Figures 7 &8. As expected, data from (NF_(*TAF*10, *FRP*2, *ALG*9)_) *versus *(NF_(*UBC*6, *TFC*1, *KRE*11)_) aligned with a good regression coefficient (panel A, R^2 ^= 0.916), while NF(_*ACT*1_) did not correlate at all with NF(_*UBC*6, *TFC*1, *KRE*11_) as shown by the worst regression coefficient of the study (panel B, R^2 ^= 0.124). Altogether, these results demonstrated the benefit of using multiple selected genes instead of a single, non-validated gene (*e.g. ACT1*) for accurate and reliable data normalization.

### Application to quantitative expression analysis of genes involved in glycogen metabolism

To test the robustness of this subset of selected reference genes, we analyzed the transcriptional regulation of genes involved in glycogen metabolism in this yeast. It has been reported that large variations of reserve carbohydrate content are associated with coordinated transcriptional regulation of the cognate genes in response to changing growth conditions or under various genetic contexts [23,24,28-33]. When examining raw Ct values from *GPH1 *(glycogen phosphorylase) and *GSY2 *(glycogen synthase) in the complete dataset (Figure 3), the very long whiskers of the boxes and numerous outliers confirmed the high variability of the expression of these two genes. On the contrary, the *SGA1 *gene encoding the vacuolar amylo-1,4 -1,6 glucosidase [34] exhibited much lower dispersion of Ct values, indicating smaller expression change than *GSY2 *and *GPH1 *under our growth conditions. Using the 3 best reference genes for data normalization (NF_(*UBC*6, *TAF*10, *ALG*9)_), we confirmed the induction of *GSY2 *and *GPH1 *between the exponential phase (S#1, calibrator sample) and the entry into the diauxic shift (S#2), and found remarkable expression ratios close to 20 for *GSY2 *and almost 100 for *GPH1 *(Figure 5). Moreover, this normalization procedure allowed us to show that the expression of these two genes dropped immediately after the diauxic shift, to return to the initial level in stationary phase for *GSY2*, or close to it for *GPH1*, while *ACT1 *normalization indicated stable and high expression of these two genes all along these growth phases. In contrast to *GSY2 *and *GPH1*, the expression of *SGA1 *showed a modest increase when cells entered the diauxic shift to reach a 3-fold activation in the stationary phase. We also analyzed for the first time transcriptional patterns in galactose-grown cells (Figure 7). Unexpectedly, the expression of *GPH1 *and *GSY2 *was already very high in the exponential phase (S#6) as compared to cultures on glucose (S#1), and it did not further increase as cells entered the diauxic shift on this carbon source, whereas glycogen accumulated with a kinetic almost similar to that on glucose (see additional files 1 &2). The expression of these two genes then dropped during the post diauxic phase to reach levels even lower than on glucose in the stationary phase. In contrast to *GPH1 *and *GSY2*, *SGA1 *expression was not affected by the carbon source. Finally, expression patterns of these three genes on galactose were the same in the CEN.PK genetic background (Figure 8) as in the KT strain (Figure 7).

The loss of *TPS1 *function had a strong impact on the glycogen accumulation pattern on galactose, as it caused hyper-accumulation of the polymer at the end of the exponential phase, and also promoted its rapid and sustained degradation during post-diauxic and stationary phases (Figure 1, and in additional file 2). Therefore, to get a preliminary idea on how this mutation could alter the glycogen kinetics at the transcriptional level, we quantified the expression of *GSY2*, *GPH1 *and *SGA1 *during growth on galactose of the WT and its *tps1 *derivative strain. It is shown in Figure 8 that the expression pattern of *GPH1 *or *GSY2 *was identical in both the wild type and in the mutant strains. The only noticeable difference was for *SGA1 *gene whose expression was already increased in the exponential phase in a *tps1 *mutant (S#14) as compared to the wild type strain (S#10, calibrator).

## Discussion

In the yeast *Saccharomyces cerevisiae*, the microarray datasets available on the *Saccharomyces *Genome Database website now represent a vast treasure-trove of reference genes suitable for gene expression normalization. We therefore used the Gene Expression Connection tool [35] to search for a set of stably expressed genes in growth dynamics [26,27]. Other approaches have recently been proposed for selection of internal controls, with statistical analysis of large microarray datasets [36,37]. Nevertheless, as stated by the authors [36,37], these *in silico *searches for stable internal controls must be accompanied by lab-bench work to verify that selected candidate genes are reliable for normalization in a specific experimental context. This is what we actually performed in the present work. Out of 13 genes analyzed in this study, *i.e*. 8 functionally unrelated genes selected from the microarray datasets together with 5 standard reference genes (*e.g*. *ACT1, PDA1 *etc), we identified *ALG9*, *TAF10*, *TFC1*, *UBC6 *and to a lesser extent *KRE11*, as the most stable genes in our experimental conditions. Another very important result from this study was the observation that geometric averaging of any subset of three genes among the six most stable genes led to very similar normalization factors, therefore highlighting the robustness of our gene selection. This conclusion was further supported by the weak expression change of this subset of genes as revealed by a survey of microarray datasets from the SGD server, *i.e*. in approximately 30 large scale transcriptomic studies, which altogether correspond to several hundreds of different samples. Therefore, *ALG9*, *TAF10*, *TFC1 *and *UBC6 *genes are the most pertinent reference genes, not only for growth phase related mRNA profiling in *S. cerevisiae*, but more generally for quantitative gene expression analysis with samples that cover a large panel of physiological and metabolic states.

Probably because of the tedious experimental validation of a suitable set of reference genes, the common practice by many authors was to use *ACT1*, *PDA1, TDH3 *or *RDN18 *as a single reference gene for normalization in *S. cerevisiae *expression studies, assuming a steady state level of expression for these so-called housekeeping genes. However, the validity of *ACT1 *as an internal standard has been already questioned [38] and *PDA1 *was preferred to *ACT1 *for normalization in Northern blot analysis since stationary phase samples showed a more significant drop of *ACT1 *mRNA. Still, by the use of Northern blot analysis, it was indeed demonstrated that *ACT1 *is a representative gene whose transcription is typically repressed following the shift from logarithmic growth to stationary phase [39,40]. As preliminary molecular clues on how down regulation of this gene occurs, these authors showed that topoisomerase I has a regulatory role in the transcriptional repression of most of the genes following the diauxic shift and in the stationary phase [39]. The second work reported a major role of the RNA polymerase II subunit RPB4, which permitted appropriate transcriptional responses during stress, including nutrient stress that accompanies entry into stationary phase [40]. Global transcriptome analysis [27] also showed that *ACT1 *mRNA levels did decrease significantly during growth in a glucose rich medium, a pattern that was confirmed by using absolute quantification by real time RT-PCR [41]. In our study, using the geometric averaging of multiple selected reference genes for relative quantification, we also found a significant drop of *ACT1 *transcripts and of other frequently used genes like *PDA1 *(E1 alpha subunit of the pyruvate dehydrogenase complex) and *IPP1 *(cytoplasmic inorganic pyrophosphatase) when cells proceed from the exponential phase of growth to the stationary phase. Therefore, as already mentioned by Monje-Cajas *et al *[41], the use of *ACT1 *and related transcripts would seriously over-estimate (*approx*. 10-fold) expression levels of genes of interest in stationary phase, leading to erroneous conclusions. Moreover, the genes encoding glycolytic enzymes, for example *TDH3 *(glyceraldehyde-3-phosphate dehydrogenase), were amongst the first yeast genes to be isolated. Because of their high expression levels, their promoters have been widely used to construct yeast expression vectors [42-47] and as model systems to study transcription [48]. Nevertheless, as details of the organisation of glycolytic promoters have emerged, it has become clear that these "simple HouseKeeping genes" actually have sophisticated molecular mechanisms controlling their expression [48]. As reviewed in this latter reference, some glycolytic enzymes were induced by glucose while others such as *ENO1 *and *TDH3 *appeared to be constitutively expressed irrespective of the carbon source (glucose or other sugars *versus *non-fermentable carbon sources). Our results, *i.e*. the large dispersion of Ct values from *TDH3 *in our experimental conditions (Figure 3) and the fact that this gene ranked amongst the worst candidate reference genes (Additional File 3), somehow contradicted this conclusion and do not encourage the use of *TDH3 *as a constitutive, internal reference gene for reliable gene normalization. Finally, the gene *RDN18 *encoding the 18srRNA could have been accepted for normalization, but the very strong expression that is several orders of magnitude above the mean expression of tested reference genes and GOIs, together with the fact that *RDN18 *does not encode mRNA precluded its use as an internal reference gene.

As an application of this normalization procedure with carefully selected reference genes, we reinvestigated in a quantitative manner the transcriptional response of genes implicated in glycogen metabolism, since this physiological event is an interesting hallmark during long term yeast cultures with significant transcriptional remodeling and huge variations of the polysaccharide content [23,24]. This study confirmed known transcriptional induction patterns of genes encoding glycogen phosphorylase (*GPH1*) and glycogen synthase (*GSY2*) that paralleled the accumulation of glycogen between the exponential phase and the diauxic shift on glucose [23,24], with a notable induction ratio of *approx*. 100-fold for *GPH1 *and 20-fold for *GSY2*. For the first time, this study also provided expression data of these genes on galactose. Despite similar growth pattern and glycogen accumulation kinetic as compared to glucose, the growth on galactose radically changed the expression pattern of these two genes. Transcripts levels were already very high in the exponential phase as compared to glucose, and we could not observe any upregulation at the entry into the diauxic-shift. Therefore, the glycogen accumulation that was observed at the end of the exponential phase during growth on galactose probably came from a concomitant activation of Gsy2p and the inactivation of Gph1p by protein phosphatases mediated dephosphorylation events [23]. With respect to the vacuolar amylo-glucosidase (*SGA1*), the large-scale transcriptional study from Gash and co-workers showed some transcriptional activation of this gene during long term yeast cultures on glucose [27,49]. Wang and co-workers [49] also provided indirect evidence of transcriptional activation of *SGA1 *in stationary phase, showing a significant role of *SGA1 *gene deletion on glycogen accumulation pattern in this phase of growth. Their result suggested that *SGA1 *may not be strictly sporulation specific, but could be activated under starvation, like in the late stationary phase. Our study supported this assertion and showed that the expression of *SGA1 *increased during the post-diauxic growth, with maximal 3-fold activation in stationary phase in wild type strains. On the other hand, the causal relationship between higher expression of *SGA1 *and faster glycogen degradation in *tps1 *derivative strains as compared to the WT remains to be investigated.

## Conclusion

A set of putative internal control genes for real-time RT-PCR analysis was selected from public microarray datasets. Using geNorm software, we validated that *ALG9*, *TAF10*, *TFC1*, *UBC6 *and to a lesser extent *KRE11*, turned out to be the most stable genes under all conditions investigated. Geometric averaging of their expression data was then applied to smooth individual variation and to calculate robust normalization factors, which allowed for the demonstration that the use of a single reference gene like *ACT1 *could lead to erroneous expression data. This set of reference genes was carefully tested in a context of large heterogeneity of samples (different physiological states during long term *S. cerevisiae *cultures, different carbon sources and genetic contexts) and applied to explore quantitatively the transcriptional regulation of genes involved in glycogen metabolism in this yeast. This study brought new insights into the transcriptional control of *GSY2*, *GPH1 *and *SGA1 *during long term growth on glucose and galactose, suggesting a potential role of *SGA1 *in the management of glycogen storage in *tps1 *cells. To summarize, this work provides a set of pertinent reference genes that should be used for validation of expression data from microarrays experiments and more generally for reliable real time RT- PCR analysis in yeast.

## Methods

### Yeast strains, growth conditions and sampling

The *Saccharomyces cerevisiae *strains, CEN.PK113-7D [50] and its *tps1 *derivative [51], and JLP48-3B (KT1112 context [24]), were grown at 30°C in a synthetic minimal medium containing 0.17% (w/v) yeast nitrogen base (DIFCO), 0.5% (w/v) ammonium sulfate and 2% (w/v) galactose (YNGal) or glucose (YNGlu). The use of prototroph strains did avoid amino acid complementation of the medium. The pH was adjusted to 5.0 with succinic acid and sodium hydroxide. Cell growth was followed by measurement of OD (600 nm) during at least 10 days. For real independency of duplicates, shake-flasks cultures were performed neither simultaneously, nor from the same inoculums. The residual extracellular carbon source was quantified by HPLC. Intracellular glycogen and trehalose were measured as described in [52]. Yeast samples for real-time PCR analysis (*approx*. 10^8 ^cells) were centrifuged (3,000 rpm, 4°C, 3 min), and the cell pellets were immediately frozen in liquid nitrogen and stored at -80°C until RNA extraction.

### Total RNA extraction

Frozen cells were mechanically disrupted using a ball mill (Mikro-Dismembrator S; B. Braun Biotech International). Total RNA was extracted using the RNeasy mini kit (Qiagen). To eliminate genomic DNA contamination, an additional DNase treatment was performed according to the RNeasy kit instruction with the RNase-free DNase set (Qiagen). The extracted RNA was quantified using the Bioanalyzer 2100 with the RNA 6000 Nano LabChip kit (Agilent) and the ND-1000 UV-visible light spectrophotometer (NanoDrop Technologies). As another preliminary quality control assay, the absence of contaminant genomic DNA in RNA preparations was verified using RNA as a template in real-time PCR assays (minus RT control, *i.e*. RNA not reverse-transcribed to cDNA).

### Quantitative RT-PCR

Oligonucleotides for real-time PCR (Table 1) were designed using Beacon Designer 2.0 software (PREMIER Biosoft International), which included a BLAST analysis against *S. cerevisiae *Genome sequence for specificity confidence, and analysis using the Mfold server to avoid positioning on risky secondary structures.

One microgram of total RNA was reverse-transcribed into cDNA in a 20 μL reaction mixture using the iScript cDNA synthesis kit (Bio-Rad). The cDNA levels were then analyzed using the MyIQ real-time PCR system from Bio-Rad. Each sample was tested in duplicate in a 96-well plate (Bio-Rad, CA). The reaction mix (25 μL final volume) consisted of 12.5 μL of iQ SYBR Green Supermix (Bio-Rad), 2.5 μL of each primer (250 nM final concentration), 2.5 μL of H_2_O, and 5 μL of a 1/10 dilution of the cDNA preparation. The absence of genomic DNA in RNA samples was checked by real-time PCR before cDNA synthesis (minus RT control). A blank (No Template Control) was also incorporated in each assay. The thermocycling program consisted of one hold at 95°C for 4 min, followed by 40 cycles of 10 s at 95°C and 45 s at 56°C. After completion of these cycles, melting-curve data were then collected to verify PCR specificity, contamination and the absence of primer dimers.

The PCR efficiency of each primer pair (Eff) was evaluated by the dilution series method using a mix of sample cDNAs as the template. Briefly, it was determined from standard curves using the formula 10^(-1/slope)^. For the calculations, the base of the exponential amplification function was used (*e.g*. 1.94 means 94% amplification efficiency). Relative expression levels were determined with efficiency correction [53], which considers differences in primer pair amplification efficiencies between target and reference genes, and results in a more reliable estimation of the "real expression ratio" than the 2ΔΔCt method [9]. Expression data and associated technical errors on duplicates were calculated using the gene expression module of the BIORAD iQ5 software, which follows models and error propagation rules outlined in the geNorm manual.

### Data analysis using geNorm

The stability of mRNA expression of tested reference genes was evaluated by using the geNorm VBA applet for Microsoft Excel [14]. This program calculates the gene expression stability measure "M" for a potential reference gene as the average pair-wise variation for that gene with all other tested genes. Then it ranks genes considering that those with the lowest M value have the most stable expression. Finally, it determines the optimal number of genes for an accurate normalization by calculating the pair-wise variation (V_(*n*/*n*+1)_) between two normalization factors, namely NF_n _(normalization factor based on the geometric mean of the n most stable genes) and NF_n+1 _: if V_n/n+1 _is superior to 0.15 as a cut-off value, one could consider that the (n+1)^th ^gene has a significant effect on normalization quality and should preferably be included for calculation of a reliable normalization factor. Authors of geNorm nevertheless recommend the minimal use of the three most stable internal control genes for calculation of the normalization factor (NF_3_), and stepwise inclusion of more control genes until the (n+1)^th ^gene has no significant contribution to the newly calculated normalization factor.

## Authors' contributions

JLP, MAT and JMF conceived the study. MAT carried out SGD microarray datasets analysis, primer design and their validation; MAT and MD carried out the cultures, samples treatment and qPCR analysis. JLP and JMF wrote the manuscript and all authors read and approved the final version.

## Supplementary Material

Additional file 1 **Growth curve and glycogen content of WT strain on glucose and galactose**. Growth (cells) and glycogen content during cultures of KT strain on glucose (set B from Figure 2) and galactose (set C from Figure 2). Cell samples (red dots) analyzed by real-time RT-PCR and sample numbering (S# followed by red numbers in the blue area). Cells (OD_600_), Glycogen (μg eq.glucose/OD unit). Click here for file

Additional file 2 **Growth curve and glycogen content of WT and *tps1 *strains**. Growth (cells) and glycogen content during cultures of CEN.PK strains on galactose, WT (set D from Figure 2) and *tps1 *(set H from Figure 2). Legend as in Additional file 1. Click here for file

Additional file 3 **Ranking of reference genes according to their expression stability**. Compiled data from all sample sets. For each set (A to M), genes are ranked from the least stable (left) to the most stable (right). The two most stable genes cannot be ranked in order. Gene expression stability value (Upper panel) as a function of gene name (lower panel). Click here for file
