# Supplementary figures and images for: Validation of reference genes for quantitative expression analysis by real-time RT-PCR in Saccharomyces cerevisiae
Source: BMC Mol Biol. 2009 Oct 30;10:99. doi: 10.1186/1471-2199-10-99 (PMC2776018; doi:10.1186/1471-2199-10-99)

## Glucose

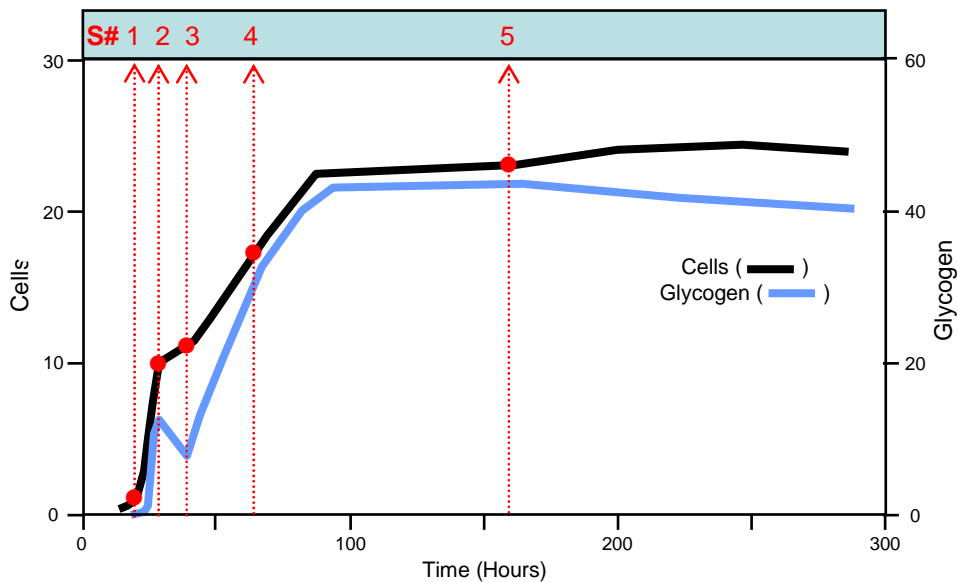

## Galactose

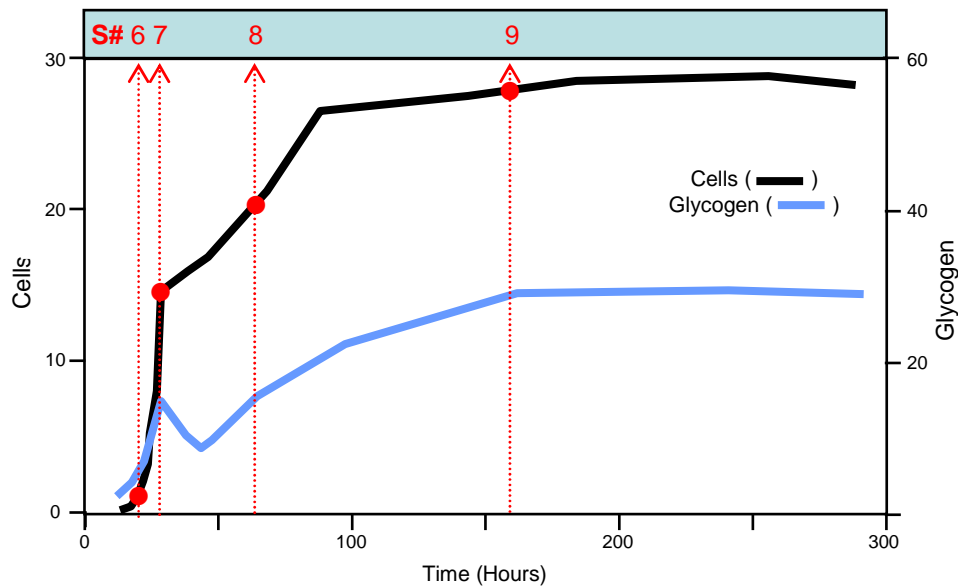

Supplement: Additional file 1 — Growth curve and glycogen content of WT strain on glucose and galactose. Growth (cells) and glycogen content during cultures of KT strain on glucose (set B from Figure 2) and galactose (set C from Figure 2). Cell samples (red dots) analyzed by real-time RT-PCR and sample numbering (S# followed by red numbers in the blue area). Cells (OD600), Glycogen (μg eq.glucose/OD unit). [file 1471-2199-10-99-S1.pdf]

WT

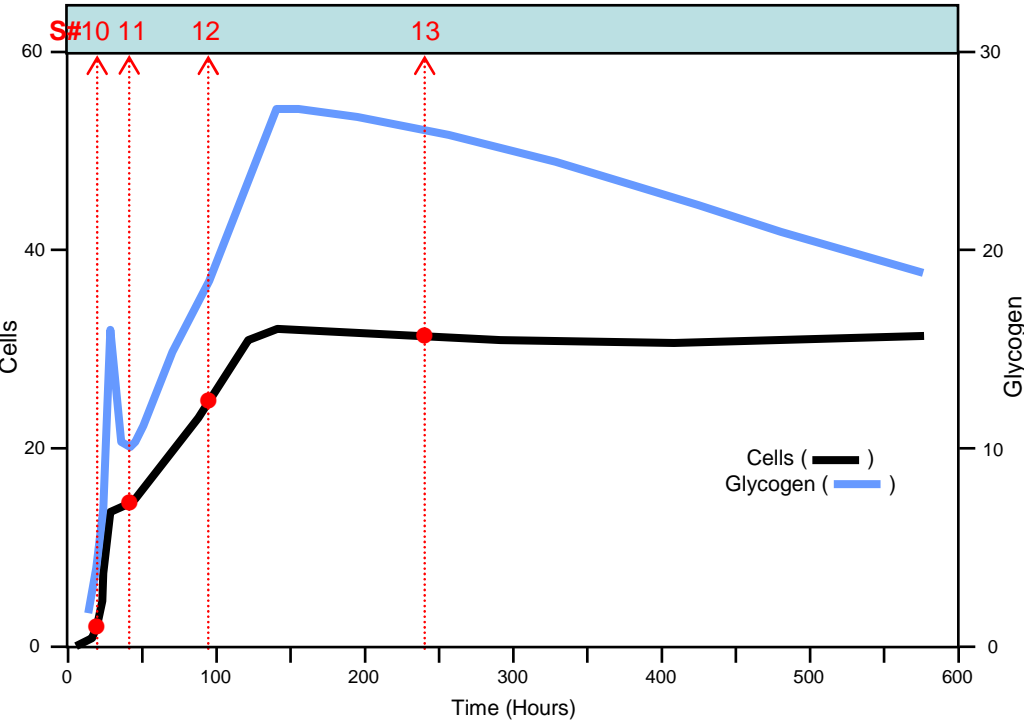

*tps1*

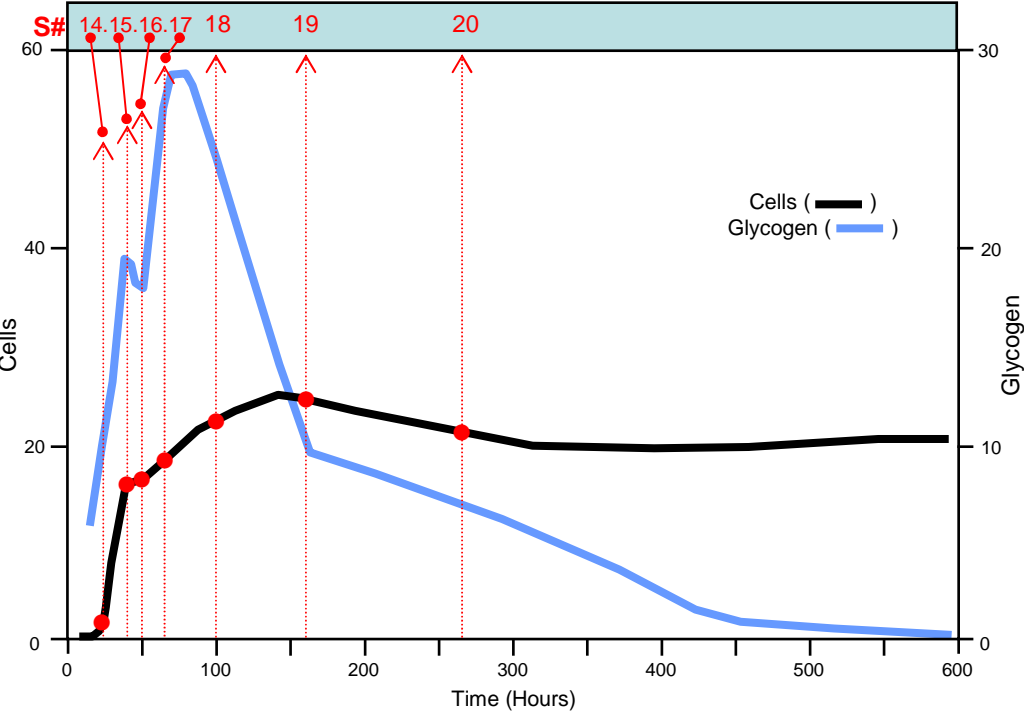

Supplement: Additional file 2 — Growth curve and glycogen content of WT and tps1 strains. Growth (cells) and glycogen content during cultures of CEN.PK strains on galactose, WT (set D from Figure 2) and tps1 (set H from Figure 2). Legend as in Additional file 1. [file 1471-2199-10-99-S2.pdf]

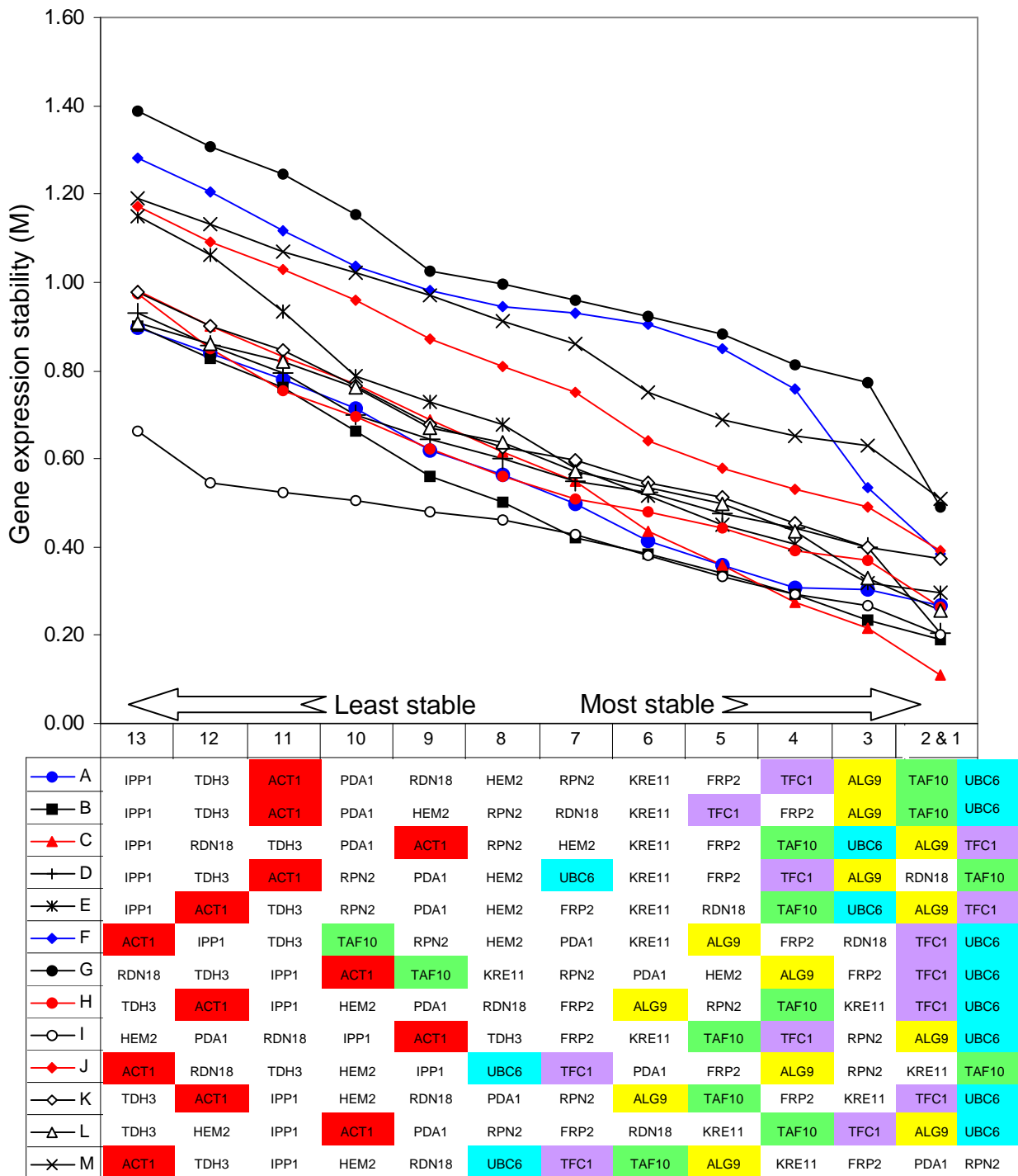

Supplement: Additional file 3 — Ranking of reference genes according to their expression stability. Compiled data from all sample sets. For each set (A to M), genes are ranked from the least stable (left) to the most stable (right). The two most stable genes cannot be ranked in order. Gene expression stability value (Upper panel) as a function of gene name (lower panel). [file 1471-2199-10-99-S3.pdf]
